# Is the Rising Birth-Rate a Blessing?

**Published:** 1920-10-16

**Authors:** 


### Is the Rising Birth-Rate a Blessing?

In his 1919 report, as Medical Officer of Health for Islington, Dr. Alfred Edwin Harris raises a topic upon which there are divergent views. There has recently been great discussion on, and inquiry into, the decline of the birth-rate of the country, that if the present increase continues—and it shows no sign of a decline in Islington—it is to be "feared" (he says) that the birth-rate will go on increasing. Recent inquiries by a Special Committee into the causes of the decline of the birth-rate of the country have clearly shown that such decline was due in a great part to the adoption of various kinds of preventive measures by parents. Various people hold different views as to whether this is good or bad for the nation; but there can be no doubt of this fact: that parents whose income is not sufficient to feed, clothe. and rear their family amidst healthy conditions should not bring children into the world to be dragged up in poverty and misery, and in dwellings which are far from wholesome and healthy.

